# Functional and Molecular Characterization of Melamine-Induced Disruption of Human Spermatozoa via Oxidative Stress and Apoptotic Pathways: An In Vitro Study

**DOI:** 10.3390/antiox15010122

**Published:** 2026-01-17

**Authors:** Francesca Paola Luongo, Eugenia Annunzi, Rosetta Ponchia, Francesca Girolamo, Giuseppe Morgante, Paola Piomboni, Alice Luddi

**Affiliations:** Department of Molecular and Development Medicine, University of Siena, 53100 Siena, Italy; francesca.luongo@unisi.it (F.P.L.); eugenia.annunzi@unisi.it (E.A.); rosetta.ponchia@gmail.com (R.P.); francesca.girolamo@student.unisi.it (F.G.); giuseppe.morgante@unisi.it (G.M.); luddi@unisi.it (A.L.)

**Keywords:** male infertility, oxidative sperm DNA damage, sperm DNA fragmentation, melamine, sperm capacitation, mitochondrial dysfunction

## Abstract

Melamine, a nitrogen-rich industrial chemical, has raised increasing concern as an emerging environmental contaminant with potential reproductive toxicity. While its nephrotoxic effects are well established, the direct impact of melamine on human sperm remains poorly defined. In this study, we investigated the in vitro effects of melamine on human sperm, under both capacitating and non-capacitating conditions. Functional analyses revealed that the exposure to 0.8 mM melamine, the highest non-cytotoxic concentration in vitro, significantly compromised sperm motility and disrupted key capacitation processes, including tyrosine phosphorylation patterns, cholesterol efflux, and the acrosome reaction. Molecular assessments demonstrated melamine-induced mitochondrial dysfunction, characterized by *COX4I1* downregulation, reduced mitochondrial membrane potential, and altered reactive oxygen species production. In parallel, gene expression analyses revealed the activation of apoptotic pathways, with the upregulation of *BAX* and downregulation of *BCL2*, changes that were more pronounced during capacitation. Furthermore, melamine exposure significantly increased sperm DNA fragmentation and denaturation, indicating genotoxic stress. Collectively, these findings demonstrate that even low, non-cytotoxic concentrations of melamine compromise sperm function by disrupting capacitation, mitochondrial activity, and genomic integrity. This study identifies capacitation as a critical window of vulnerability and underscores the need to consider melamine as a potential environmental risk factor for male reproductive health.

## 1. Introduction

Over the past several decades, male idiopathic infertility has significantly increased, a trend that has been linked to rising industrialization and the widespread release of synthetic pollutants into the environment [1,2]. Among these, melamine has emerged as a significant concern due to its toxicological impact when misused or introduced into the food chain. Melamine (2,4,6-triamino-1,3,5-triazine; CAS: 108-78-1) is a nitrogen-rich heterocyclic compound commonly used in industrial manufacturing and formulation processes. Its high nitrogen content, comprising approximately 66% of its molecular weight, allows it to artificially inflate protein readings in food, leading to its deliberate manipulation in protein-based products [3]. Currently, melamine is under assessment as an endocrine disruptor chemical by the European Chemical Agency (ECHA) (https://echa.europa.eu/it/substance-information/-/substanceinfo/100.003.288, accessed on 1 October 2025). Toxicokinetic studies in rats have shown that melamine is rapidly absorbed (73–98%) and distributed throughout the body within 15 min of oral administration [4]. The highest tissue concentrations are typically found in the kidneys and urinary bladder, consistent with its well-established nephrotoxicity. However, appreciable levels have also been reported in the liver, gastrointestinal tract, spleen, heart, lungs, brain, and reproductive organs. While early research has primarily addressed melamine-induced nephrotoxicity and hepatic alterations [5], emerging evidence indicates that the reproductive system—highly sensitive to environmental toxicants—may also represent a major target of toxicity [6]. Notably, melamine exposure has been linked to disruption of the blood–testis barrier [7], increased sperm abnormalities, and DNA damage [8]. In murine models, testicular damage has been associated with oxidative stress and apoptosis pathways [9]. However, the full spectrum of melamine’s impact on male fertility remains poorly characterized. In particular, little is known about its direct effects on sperm under controlled in vitro conditions, where confounding systemic factors can be excluded. Given the global concern over declining male fertility rates and the rise in environmental pollutants as contributing factors, understanding the effects of melamine on sperm function is crucial for public health. This study was therefore designed to evaluate the in vitro effects of different melamine concentrations on human spermatozoa, both before and during capacitation, a key physiological process required for the acquisition of fertilizing ability. Beyond assessing sperm vitality and motility, we explored the underlying molecular mechanisms potentially affected by melamine through the analysis of hormone receptors and apoptotic effectors gene expression. Furthermore, mitochondrial function and oxidative stress were evaluated to gain deeper insight into how melamine may compromise sperm competence.

## 2. Materials and Methods

### 2.1. Participants

The study was conducted on a group of men (aged 18–44 years) who were attending the Medically Assisted Reproductive Unit at Siena University Hospital for fertility assessment and semen analysis. The study was approved by the Ethical Committee of the Siena University Hospital (CEAVSE, protocol no. 18370, dated 2 October 2020), and written informed consent was obtained from all participants. A detailed clinical history was collected, and individuals with known causes of male infertility, including endocrine disorders, varicocele, or cryptorchidism, were excluded. Furthermore, all participants had a normal karyotype and BMI within the normal range (18.5–24.9 kg/m^2^), no history of chemotherapy, radiotherapy, or chronic illnesses.

### 2.2. Semen Analysis

Semen samples were collected by masturbation following 3–5 days of sexual abstinence. After complete fluidification at room temperature, semen analysis was conducted according to 2021 WHO guidelines [10]; to ensure objectivity, the analysis was conducted by two distinct, blinded observers, with the final reported values reflecting the mean of their observations. The volume, viscosity, pH, and appearance of the semen were evaluated together with sperm concentration, progressive and total motility, and morphology. Sperm concentration and motility were evaluated by loading 10 µL of well-mixed semen into a Makler counting chamber and observing under an optical microscope (Nikon, Nikon Europe B.V., Amsterdam, the Netherlands) at 200× magnification. Total sperm, motile, and non-motile sperm were evaluated. Sperm morphology was evaluated by using pre-colored glasses (Testsimplets), whereas sperm vitality was assessed using eosin staining [11]. To this end, 10 µL of semen sample and 10 µL of eosin Y solution (7.2 mM) were mixed and examined under a light microscope at 400× magnification. Non-viable sperm absorbed the dye and appeared pink, whereas live sperm excluded stain and remained colorless. Sperm concentration, motility, morphology, and vitality were assessed for each sample, and only those individuals with normal sperm parameters (n = 20) were included in the study.

### 2.3. In Vitro Sperm Treatment with Melamine

Following liquefaction, semen samples were processed using standard laboratory procedures, and analyses were performed on the total sperm fraction, without further separation into morpho-functional subpopulations. In detail, semen samples were washed with Dulbecco’s phosphate-buffered saline without calcium and magnesium (DPBS, Corning, NY, USA) and then centrifuged at 800× *g* for 10 min. After that, the seminal plasma was removed, and sperm were resuspended in DPBS. To ensure correct normalization, the sperm concentration was then redetermined, and aliquots, each containing 40 million sperm, were prepared and resuspended in either DPBS (to maintain the uncapacitated state) or in capacitating medium (to induce capacitation). The capacitation medium consisted of 20 mM 4- (2-Hydroxyethyl) piperazine-1-ethanesulfonic acid (HEPES), 112 mM NaCl, 3.1 mM KCl, 5 mM glucose, 21.7 mM sodium L-lactate, 1 mM sodium pyruvate, 0.3 mM Na_2_HPO_4_, 0.4 mM MgSO_4_ × 7H_2_O, 4.5 mM CaCl_2_ × 2H_2_O, 5 mg/mL BSA and 15 mM bicarbonate; pH = 7.4 ± 0.1; osmolality = 0.300 Osm/Kg ± 0.01. Prepared aliquots were divided into control and Melamine-treated samples (0.8 mM final concentration) under both basal (uncapacitating) or capacitating conditions. In this way, each sample serves as its own control under melamine-treated and untreated conditions. Samples were incubated for 90 min at 37 °C with 5% CO_2_. At the end of the incubation, samples were centrifuged at 600× *g* for 10 min, and the resulting pellets were used for subsequent analyses.

### 2.4. Immunofluorescence and Acrosomal Staining

Immunofluorescence analysis was performed according to a previously published protocol [12]. Initially, sperm samples were washed, smeared onto glass slides, and fixed in 4% paraformaldehyde for 15 min. Cell permeabilization was carried out using 0.5% Triton X-100 in PBS for 5 min. Then, sperm were incubated with 5% bovine serum albumin (BSA)/1% normal goat serum in PBS for 30 min to prevent non-specific binding. Samples were then incubated overnight at 4 °C with the primary mouse anti-human P-Tyrosine (P-Tyr) antibody diluted in 1% NGS and 1% BSA in PBS. After three PBS washes the secondary antibody was applied for 1h at RT (see Supplementary Table S1). After additional 3 PBS washes, sperm were incubated for 30 min with 30 µg/mL fluorescein isothiocyanate-labeled Pisum Sativum agglutinin (FITC-PSA) to label the acrosome. Following 10-min PBS wash, nuclei were counterstained with DAPI, and slides were mounted using DABCO antifade solution. Imaging analysis was performed using a Leica AF6500 Integrated System for Imaging and Analysis (Leica Microsystems, Wetzlar, Germany) equipped with the LAS software (LAS X Life Science). P-Tyr staining patterns were classified according to published criteria [13,14], and the percentages of acrosome-reacted and intact acrosome sperm were determined.

### 2.5. Western Blot Analysis

Western blotting was performed according to validated procedures [13]. Briefly, 50 μg of total denatured proteins were separated on 8% polyacrylamide gel, and then transferred to a nitrocellulose membrane (GE Healthcare, Chicago, IL, USA). Non-specific binding was blocked by incubating the membrane for 1h in 5% nonfat dry milk; after that, an overnight incubation was performed at 4 °C with primary antibodies (see Supplementary Table S1), diluted in 1% non-fat dry milk/TTBS (TBS containing 0.2% Tween 20). After the washes in TTBS, the membrane was incubated with the corresponding horseradish peroxidase (HRP)-conjugated secondary antibody (see Supplementary Table S1). The resulting immunoreactive bands were detected using chemiluminescence with ImageQuant LAS 4000 (GE Healthcare), and the relative intensity of the bands was determined by densitometric analysis using Image J (1.54p).

### 2.6. Chlortetracycline Fluorescence Assay

The chlortetracycline (CTC) fluorescence assay was used to detect Ca^2+^-related changes associated with sperm capacitation. On the day of analysis, a working CTC solution was prepared at 750 µM (Sigma, St. Louis, MO, USA) in buffer containing 130 mM NaCl, 5 mM cysteine, and 20 mM Tris-HCl, adjusted to pH 7.8. To prevent photodegradation, the solution was wrapped in aluminum foil and maintained at 4 °C until use. For each condition, spermatozoa were placed on microscope slides, fixed with 4% paraformaldehyde (10 min), and washed twice with PBS. A 100 µL aliquot of the CTC solution was then applied to each slide and incubated for 10 min in the dark. After incubation, slides were rinsed with PBS to remove residual dye, overlaid with 40 µL of DABCO, and sealed with coverslips. Samples were visualized using a Leica DMB 6000 fluorescence microscope, and image acquisition was carried out with a CTR6500 HS digital camera (Leica Microsystems, Wetzlar, Germany).

### 2.7. RNA Extraction, Retrotranscription and qPCR

Total RNA was extracted using QIAzol Lysis Reagent (QIAGEN, Germantown, MD, USA) as previously described [14]. Each sample was mixed with 1 mL of QIAzol and 200 µL of chloroform, then centrifuged at 12,000 rcf for 15 min at 4 °C. The aqueous phase was collected, mixed with an equal volume of cold isopropanol, incubated for 30 min at 4 °C, and centrifuged again. The RNA pellet was washed twice with 75% ethanol and resuspended in nuclease-free water. RNA quality was determined with NanoDrop™ One/OneC spectrophotometer (Thermo Scientific™, Waltham, MA, USA). After that, 500 ng of RNA was reverse-transcribed using the High-Capacity cDNA Reverse Transcription Kit (Thermo Fisher Scientific Inc., Waltham, MA, USA). qRT-PCR was performed using Quant Studio 3 (Thermo Fisher Scientific Inc., Waltham, MA, USA) and specific primers (Supplementary Table S2), in 10 µL reactions containing 5 µL of PowerTrack™ SYBR Green Master Mix, 1 µM of each primer, and 3 µL of nuclease-free water. The thermal protocol included 95 °C for 10 min, followed by 45 cycles at 95 °C for 3 s and primer-specific annealing (see Supplementary Table S2). Reference gene stability was assessed with RefFinder-master (https://www.ciidirsinaloa.com.mx/RefFinder-master/?type=reference, 1 September 2025), identifying *GAPDH* and *B2M* as the most stable. Expression levels were normalized to these genes and analyzed using the 2^−ΔΔCt^ method, with results expressed relative to a calibrator sample, identifying 18S as the most stable, followed by GAPDH, and B2M as the least stable; thus, gene expression analyses were normalized using 18S and GAPDH.

### 2.8. JC-1 for Assessing Mitochondrial Membrane Potential (MMP)

Alterations in MMP of spermatozoa under non-capacitating and capacitating conditions treated with 0.8 mM of melamine were evaluated using the cation 5,5′,6,6′-tetrachloro-1,1′,3,3′-tetraethylbenzimidazolcarbocyanine iodide (JC-1) dye (Molecular Probes, Eugene, OR, USA) [14]. Sperm were incubated with 1 μg/mL JC-1 dye diluted in PBS for 20 min at 37° C, in the dark. MMP levels were evaluated using a Leitz Aristoplan fluorescence microscope (Leica, Wetzlar, Germany) with a 490 nm excitation light and a 530 nm barrier filter. For each sample, at least 300 sperm were analyzed at 630× magnification. Sperm with high MMP showed red fluorescence at the midpiece due to JC-1 aggregate formation. Conversely, sperm with low MMP exhibited a diffuse green signal, indicating JC-1 in its monomeric form. Results are expressed as a percentage red/green ratio.

### 2.9. ROS Detection

Intracellular ROS were determined using the 2′,7′-dichlorodihydrofluorescein diacetate dye (DCF) (Life Technologies Corp., Carlsbad, CA, USA). Sperm were incubated with DCFH-DA solution (10 μM in PBS) at 37 °C for 15 min. Following incubation, the samples were centrifuged to allow for the collection of the supernatant. Fluorescence intensity was measured using excitation and emission wavelengths of 480 and 520 nm, respectively, using Synergy HTX multi-mode reader (BioTek, Winooski, VT, USA) and normalized to 30 × 10^6^ sperm cells.

### 2.10. DNA Fragmentation Analysis

Sperm DNA integrity and compaction were evaluated using the acridine orange (AO) test, conducted according to established protocols. For each sample, three replicates were prepared on glass slides, air-dried, and fixed in freshly prepared Carnoy’s solution (methanol/glacial acetic acid, 3:1) for at least three hours or overnight. After air-drying, slides were incubated in a buffer (80 mmol/L citric acid, 15 mmol/L Na2HPO^4^, pH 2.5) at 75 °C for five min. Slides were then incubated for 5 min in freshly prepared AO solution (2.5 mL of 1% AO stock, 10 mL 0.1 mol/L citric acid, 400 µL 0.3 mol/L Na2HPO^4^·H_2_O). After a washing step, slides were wet-mounted and examined using a Leica AF6500 Integrated System for Imaging and Analysis (Leica Microsystems, Wetzlar, Germany) equipped with the LAS software(LAS X Life Science). Sperm with intact DNA exhibited a green signal, whereas those with decompacted/damaged DNA showed yellow-green to red fluorescence. The percentage of intact chromatin was determined by counting 100–200 sperm per slide. To ensure accuracy and reduce bias, each slide was independently analyzed by two independent observers, and the mean value was used for analysis.

Sperm DNA fragmentation was assessed using the TUNEL assay (DeadEnd™ Fluorometric TUNEL System (Promega, Madison, WI, USA), following the manufacturer’s instructions. Briefly, semen samples were washed with DPBS and centrifuged at 600× *g* for 10 min. After centrifugation, the pellet was resuspended in DPBS at a concentration of 10 × 10^6^ sperm/mL. The sample was then smeared onto a slide, fixed in 4% paraformaldehyde for 25 min, washed with 1% BSA/DPBS and treated with 0.2% Triton^®^ X-100 for 5 min, followed by two additional washes. The slides were then incubated with an equilibration buffer for 10 min at RT, followed by incubation with the reaction mixture containing rTdT enzyme, nucleotide mix, and equilibration buffer at 37 °C for 1 h. After incubation, the slides were immersed in 2× SSC for 15 min and washed three times (5min each) in 1% BSA/DPBS to remove unincorporated fluorescein-12-UTP. The slides were counterstained with DAPI and mounted with DABCO. Images were acquired using a Leica DM6000 fluorescence microscope. For negative controls, the TUNEL reaction mixture was substituted with the label solution. Sperm were categorized based on their fluorescence profile: cells exhibiting blue staining were identified as having intact DNA, while those displaying bright green fluorescence were classified as having significant DNA fragmentation.

### 2.11. Statistical Analysis

All analyses were conducted using GraphPad Prism 8 for Windows (v8.0.2; Dotmatics, Boston, MA, USA), with *p*-values < 0.05 considered statistically significant. Data are expressed as mean ± standard deviation (SD). The statistical significance between control and treated groups was assessed using two-way ANOVA, considering treatment (melamine concentration) and incubation condition (capacitating vs. non-capacitating) as independent factors. To control for Type I error in predefined pairwise comparisons, Bonferroni’s multiple comparison post hoc tests were applied. One-way ANOVA followed by Tukey’s multiple comparison test was used for Western blot analyses to compare protein abundance among multiple experimental groups.

## 3. Results

### 3.1. Cytotoxic and Biological Effects of Melamine on Sperm Capacitation and Fertilizing Ability

**Figure 1:**
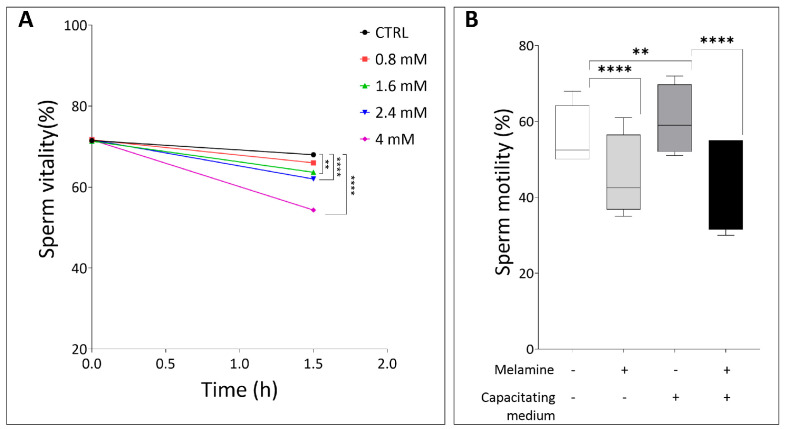
(**A**) Sperm vitality at different timepoints (in hours), comparing control and melamine-treated samples. (**B**) Percentage of sperm motility in basal or in capacitating conditions, with (+) and without (−) melamine (0.8 mM). Significant differences are indicated with Bonferroni correction ** *p* < 0.01; **** *p* < 0.0001.

To assess the effects of melamine, only semen samples conforming to the WHO [10] reference standards for normozoospermia were included, thereby minimizing bias due to inter-sample variability in sperm parameters. The associated seminal characteristics are presented in Supplementary Table S3. Sperm samples were in vitro exposed to increasing concentrations of melamine, selected according to reference data on human exposure levels. In the general population, urinary melamine levels are typically low, usually remaining below 25 µg/g creatinine (about 0.4 µM). Levels exceeding 5µM melamine have been identified as indicators of significant point-source exposure, such as consumption of contaminated food or leaching from melamine-based tableware [15,16]. Based on these data, spermatozoa were treated in vitro with increasing melamine concentrations (0.8, 1.6, 2.4, and 4 mM) that are well above the proposed safety threshold, and sperm vitality was subsequently assessed. No significant decrease in sperm vitality was observed at 0.8 mM melamine compared to the control group. However, exposure to concentrations above 1.6 mM caused a statistically significant decline in sperm vitality (*p* < 0.05), indicating a dose-dependent cytotoxic effect (Figure 1A).

Based on these findings, 0.8 mM melamine was selected as the highest non-cytotoxic concentration for subsequent experiments. Interestingly, despite not affecting sperm viability, this dose significantly impaired motility under both capacitating and non-capacitating conditions compared to controls (*p* < 0.0001, Figure 1B). These findings demonstrate that even concentrations of melamine that are not directly cytotoxic can adversely affect sperm functional parameters critical for fertilization.

**Figure 2:**
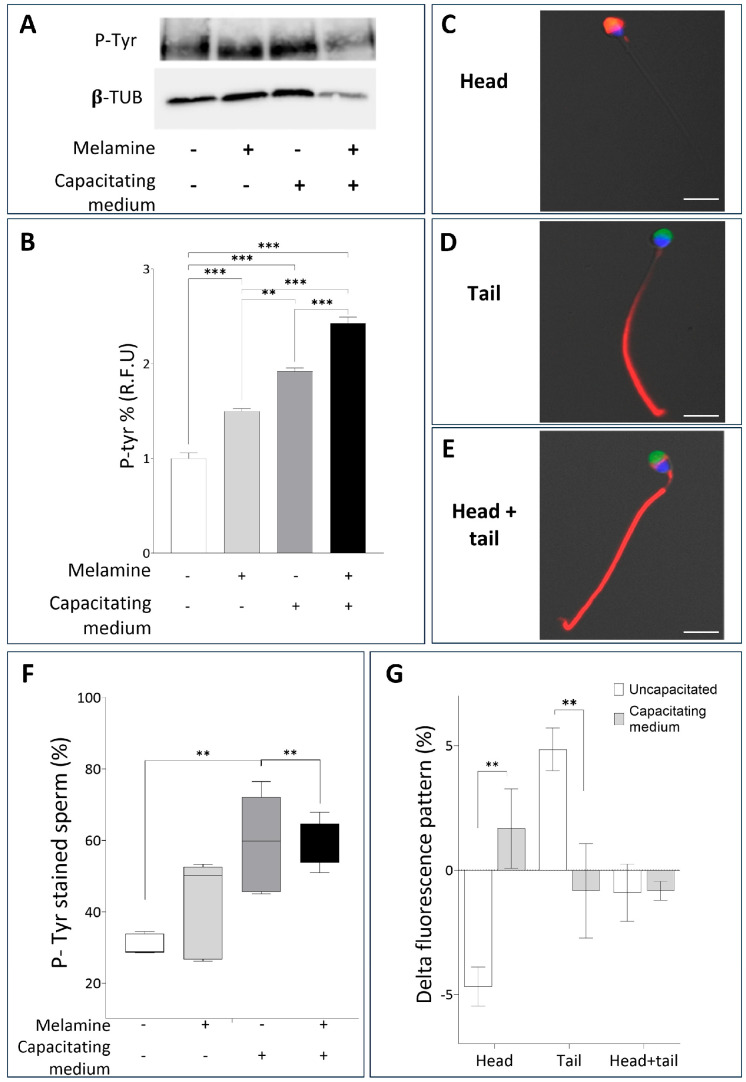
(**A**) Representative Western blot analysis of P-Tyr in human sperm not treated (−) or treated with melamine (+) under capacitating (+) or non-capacitating (−) conditions. β-tubulin was used as a loading control. (**B**) Bar graph showing the relative fluorescence unit (RFU) of P-Tyr bands, quantified by computer-assisted densitometric analysis. Data show the means ± SD of relative units (RU). (C-E) Representative immunofluorescent micrographs of human sperm illustrating the three different patterns monitored: (**C**) head, (**D**) tail and (**E**) head-tail, evaluated by P-Tyr localization (red) and PSA staining (green); nuclei were counterstained in blue. Bar = 10 µm. (**F**) Whisker-plot graph showing the percentage of P-Tyr–positive spermatozoa after melamine exposure under non-capacitating or capacitating conditions. Boxes show the interquartile range with median and mean values, and whiskers represent min and max confidence intervals. (**G**) Bar chart showing the distribution of P-Tyr signal in melamine-treated sperm. P-Tyr–positive cells were classified by fluorescence localization (head, tail, head + tail). Percentages were normalized to controls and expressed as Δ. Significance ** *p* < 0.01, *** *p* < 0.001.

We next examined whether melamine affects molecular events of capacitation, focusing on tyrosine phosphorylation, a post-translational modification essential for regulating capacitation and the acquisition of fertilizing competence. The Western blot comparison of tyrosine-phosphorylated protein levels between the three study groups is shown in Figure 2A, where a band of about 200 kD corresponding to the family of proteins with tyrosine-phosphorylated residues is clearly visible. The quantification of P-Tyr relative abundance revealed a significant melamine-induced increase, under both non-capacitating and capacitating conditions (Figure 2B). Since our experimental design did not include procedure for sperm selection, the measured signal could reflect the phosphorylation level within individual sperm as well as the proportion of spermatozoa undergoing phosphorylation. To clarify this, we assessed the percentage of P-Tyr-positive spermatozoa using immunolocalization of P-Tyr, also identifying the relative frequency of specific staining patterns (Figure 2C–E). As shown in Figure 2F, melamine treatment significantly increased the percentage of sperm positive for P-Tyr residues, with a stronger effect under non-capacitating conditions (*p* < 0.01). These findings suggest that melamine may alter the early stages of sperm capacitation (Figure 2F). To better characterize the P-Tyr signal distribution, we categorized positively stained spermatozoa according to fluorescence localization: head, tail, or both. For each group, the number of P-Tyr-positive sperm in melamine-treated samples was normalized to the corresponding control and expressed as percentage differences. Under capacitating conditions, melamine-treated sperm showed a significant increase in head-localized P-Tyr signal compared to the uncapacitated group (*p* < 0.01). Conversely, sperm exposed to melamine under capacitating conditions exhibited a significant decrease in tail-localized P-Tyr signal compared to non-capacitating conditions (*p* < 0.01). No significant changes were observed in sperm with combined head and tail localization. (Figure 2G). Considering that tyrosine phosphorylation in the principal piece of the flagellum is a specific capacitation-associated event [17] this shift in localization suggests that melamine disrupts the compartmentalized signaling events essential for proper capacitation and hyperactivated motility.

**Figure 3:**
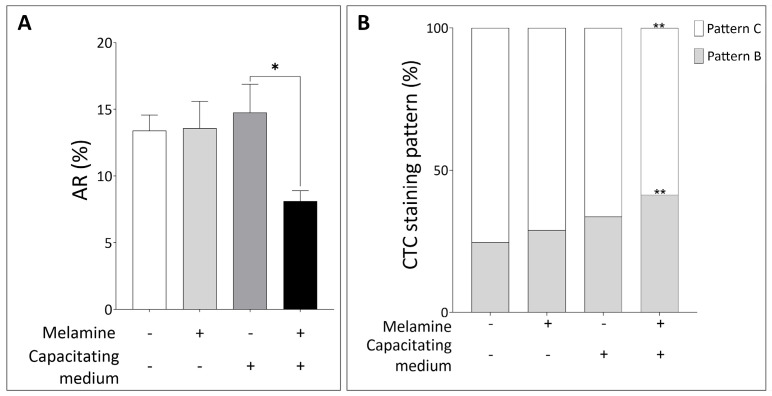
(**A**) Percentage of Acrosome Reaction (AR) and (**B**) CTC staining pattern in sperm not treated (−) or treated with melamine (+) under capacitating (+) or non-capacitating (−) conditions. Pattern B: capacitated, acrosome intact sperm. Pattern C: capacitated, acrosome-reacted sperm. Significant differences are indicated with Bonferroni correction * *p* < 0.05; ** *p* < 0.01.

Furthermore, given that head-localized P-Tyr is typically less abundant in sperm undergoing the acrosome reaction [17], we next analyzed the acrosomal status. As shown in Figure 3A, melamine exposure under capacitating conditions significantly reduced the proportion of acrosome-reacted sperm, which aligns with the increased head-localized P-Tyr observed in treated samples. This result was further validated by CTC staining: melamine treatment led to a significant rise in the percentage of capacitated sperm with intact acrosome (pattern B) under capacitating conditions (*p* < 0.05). At the same time, the proportion of capacitated acrosome-reacted sperm (pattern C) was significantly reduced, in agreement with PSA staining results (*p* < 0.05, Figure 3B).

### 3.2. Alteration of Mitochondrial Function and Related Molecular Pathways

**Figure 4:**
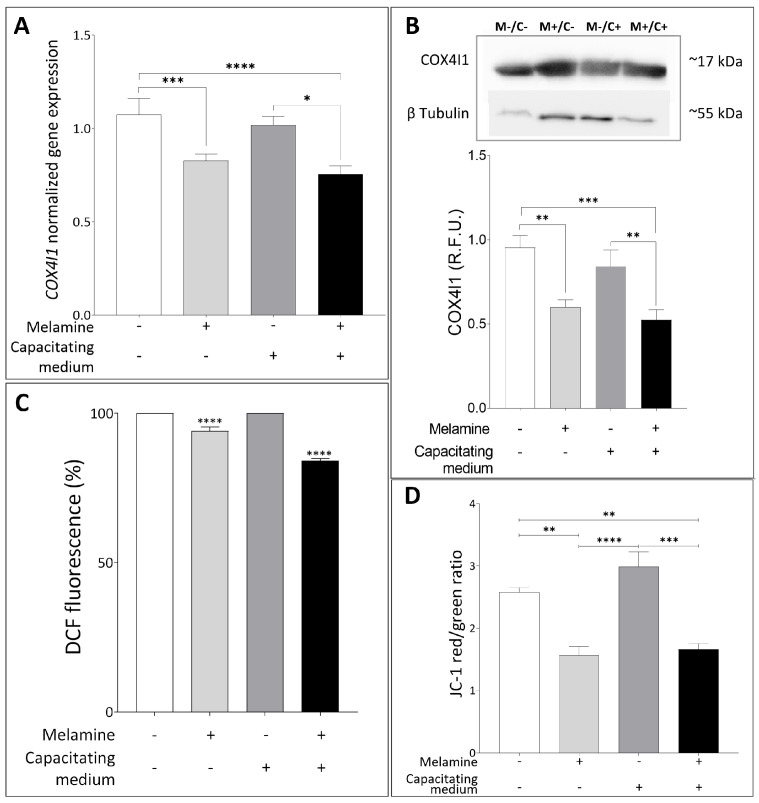
(**A**) Normalized gene expression of *COX4I1* between not treated (−) or treated with melamine (+) under capacitating (+) or non-capacitating (−) conditions, calculated by Delta–Delta Ct (ΔΔCt) method. Significant differences are indicated with Bonferroni-corrected *p* value: * *p* < 0.05; *** *p* < 0.001; **** *p* < 0.0001. (**B**) Representative Western blot analysis of COX4I1 in human sperm exposed (M+) or not-exposed (M−) to melamine under capacitating (C+) or non-capacitating (C−) conditions; β-tubulin was used as a loading control. At the bottom, bar graph shows the relative fluorescence unit (RFU) of COX4I1, quantified by computer-assisted densitometric analysis and normalized against the control. ** *p* < 0.01; *** *p* < 0.001. (**C**) Percentage of Dichlorofluorescein (DCF)-stained sperm between control and treatment with melamine normalized to 30 × 10^6^ sperm cells. **** *p* < 0.0001. (**D**) Bar plot graph showing JC-1 red (high MMP)/green (low MMP) ratio. Significant differences are indicated with Bonferroni-corrected *p* value: ** *p* < 0.01; *** *p* < 0.001; **** *p* < 0.0001.

To better understand how melamine impairs human sperm function, we focused on mitochondria as a key regulator of sperm energy metabolism, driving motility and capacitation process, and well-recognized targets of environmental toxicants [18]. In line with this, we examined the expression of *COX4I1*, a gene critical for oxidative phosphorylation and the regulation of reactive oxygen species (ROS). Exposure to 0.8 mM melamine resulted in a significant downregulation of *COX4I1* expression, under both basal and capacitating conditions (*p* < 0.0001) (Figure 4A). This finding was corroborated at the protein level by Western blot analysis, which revealed a marked reduction in COX4I1 abundance following melamine treatment (Figure 4B). Together, these results suggest that melamine may compromise mitochondrial function and energy metabolism, thereby compromising sperm fertilizing potential.

Since alterations in COX4I1 expression or function may disturb the redox balance [19,20], we further investigate the intracellular ROS amount by using DCF staining. A significant reduction in ROS levels was detected in sperm samples treated with melamine under both non-capacitating and capacitating conditions, suggesting that melamine may impair physiological ROS production, essential for normal sperm function (Figure 4C). These findings led us to examine mitochondrial function more closely; therefore, we assessed changes in mitochondrial membrane potential (MMP) by measuring the JC-1 red/green fluorescence ratio in the sperm midpiece. As shown in Figure 4D, melamine induced a significant reduction in this ratio, indicative of low, depolarized membrane potential, both in basal and capacitating conditions (*p* < 0.05 and *p* < 0.01, respectively). These combined findings indicate that melamine exposure disrupts mitochondrial activity, which may contribute to impaired sperm function through altered redox balance and early apoptotic signaling pathways, consistent with the cytotoxic effect seen at higher melamine concentrations.

**Figure 5:**
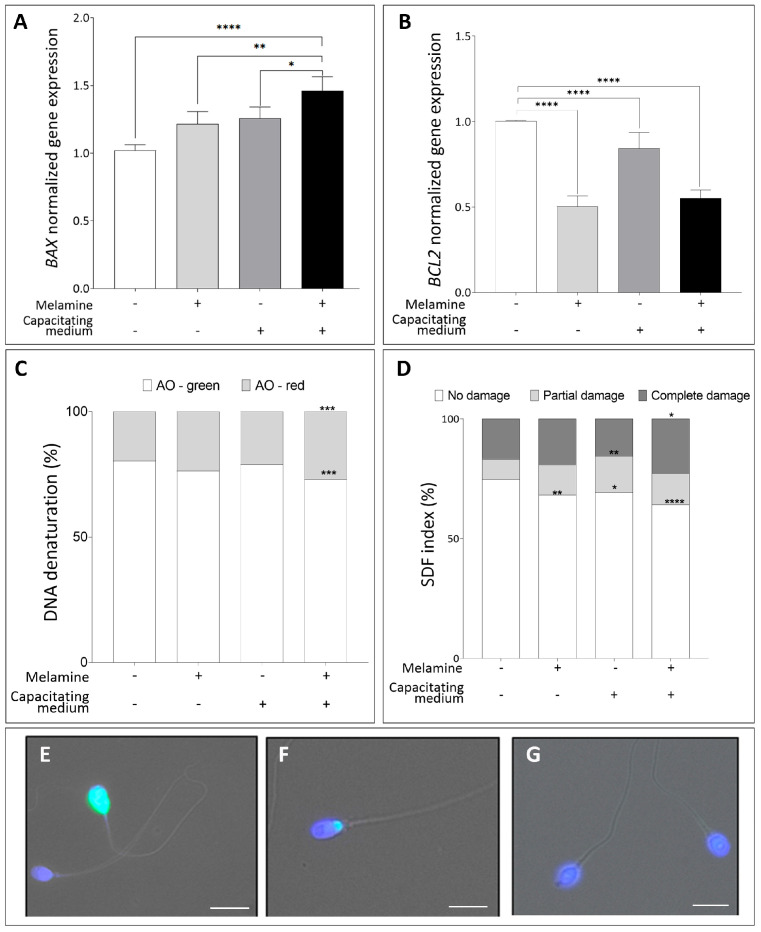
(**A**,**B**) Normalized gene expression of *BAX* (**A**), *BCL2* (**B**) in control (-) versus melamine-treated (+) sperm with (+) or without (-) capacitating media, calculated using ΔΔCt method. Significant differences are indicated with Bonferroni-corrected *p* value: * *p* < 0.05; ** *p* < 0.01; **** *p* < 0.0001. (**C**) Percentage of DNA denaturation classified as sperm with normally condensed non-fragmented chromatin (green) and decondensed or damaged chromatin (red). *** *p* < 0.001. (**D**) TUNEL assay for sperm DNA fragmentation showing the percentage distribution of sperm showing no damage, partial damage, or complete DNA damage. Significant differences are indicated by Bonferroni-corrected *p* value: * *p* < 0.05; ** *p* < 0.01; **** *p* < 0.0001. (**E**,**F**) Representative fluorescence images of the different patterns identified for specific patterns: complete damage (**E**), partial damage (**F**), no damage (**G**), Bar = 10 µm.

To further investigate this possibility, we assessed the expression of key apoptosis-related genes—*BAX* and *BCL2*—after in vitro exposure to melamine. As shown in Figure 5A, *BAX* expression was significantly upregulated in melamine-treated capacitated sperm compared to all other groups (*p* < 0.01 and *p* < 0.0001), reflecting an enhanced apoptotic response during capacitation. In contrast, the anti-apoptotic gene *BCL2* was significantly downregulated in melamine-exposed sperm under both conditions, with a more pronounced reduction observed during capacitation (*p* < 0.0001), suggesting a shift in the *BAX*/*BCL2* ratio in favor of apoptosis (Figure 5B).

To further assess the genotoxic potential of melamine on sperm DNA, we evaluated the DNA denaturation and fragmentation using the AO staining and TUNEL assay. As illustrated in Figure 5C, melamine treatment caused a pronounced increase in DNA denaturation specifically during capacitation, evidenced by a significant shift from AO-green (intact DNA) to AO-red (denatured DNA) in melamine-exposed capacitated sperm (*p* < 0.001) compared to non-capacitated control, whereas non-capacitated samples showed no significant changes. Moreover, exposure to melamine significantly increased the sperm DNA fragmentation index (SDF) (Figure 5D) in both non-capacitated and capacitated sperm. These results were fully consistent with the AO staining results confirming the impact of melamine on sperm DNA integrity.

## 4. Discussion

This study demonstrates that melamine, even at non-cytotoxic concentrations, exerts a significant detrimental impact on human sperm function. Specifically, melamine exposure impairs motility, disrupts the molecular events associated with capacitation, reduces the acrosome reaction, induces early mitochondrial dysfunction and apoptotic responses. These effects were consistently observed under both non-capacitating and capacitating conditions, with a greater magnitude of disruption evident during capacitation. This suggests that melamine interferes with the signaling networks required for the acquisition of fertilizing competence.

Previously, in vivo studies in animal models have reported that chronic or high-dose melamine exposure causes testicular damage, sperm abnormalities, and impaired fertility [21,22]. These effects have been linked to oxidative stress, disruption of energy metabolism, and apoptosis in testicular cells, ultimately impairing sperm motility [23].

Our results extend these observations to human spermatozoa in vitro. In our model, the use of 0.8 mM melamine serves as a positive signal to identify the biochemical targets of the toxin, acknowledging that in vitro sensitivity often differs from in vivo complexity where cumulative low-dose effects occur over a 74-day spermatogenic cycle.

However, given that human spermatozoa are terminally differentiated cells with a limited lifespan post-ejaculation—where prolonged incubation introduces confounding oxidative damage unrelated to the toxin—this reductionist approach was necessary to identify direct cellular susceptibilities and mechanistic pathways within a controlled, biologically relevant timeframe.” Indeed, by employing this concentration, we demonstrated that even doses, which do not cause overt cytotoxicity, are sufficient to compromise sperm motility, likely through similar mechanisms involving mitochondrial dysfunction and redox imbalance. Indeed, we observed a melamine-induced downregulation of *COX4I1* expression, the subunit of cytochrome c oxidase essential for oxidative phosphorylation [24], together with a reduction in MMP, indicating impaired electron transport chain activity. Mitochondrial dysfunction induced by melamine is expected not only to limit ATP availability, thereby reducing the energy required for flagellar movement [25], but also to alter the physiological production of ROS associated with mitochondrial activity. ROS play a dual role in spermatozoa, acting as essential signaling molecules for capacitation at physiological levels, yet becoming detrimental when dysregulated [26,27].

Our results show a reduction in ROS in melamine-treated human sperm, in contrast with previous in vivo animal studies reporting increased oxidative stress in the testis following melamine exposure [28]. This highlights the physiological role of ROS in sperm, where basal levels are required to trigger capacitation, support hyperactivated motility, and prime the acrosome reaction. P-Tyr, particularly in the flagellar principal piece, is a hallmark of capacitation and is closely linked to hyperactivated motility [29,30,31]. In melamine-treated sperm, we observed an overall increase in global P-Tyr levels; however, this did not translate into a higher proportion of capacitated cells. Rather, there was a pronounced redistribution of P-Tyr staining toward the sperm head, a pattern previously associated with defective capacitation or premature acrosome reaction [32]. The accumulation of P-Tyr residues in the head, coupled with their reduction along the flagellum, indicates that melamine disrupts the normal spatial progression of capacitation, potentially triggering premature or dysregulated activation of acrosome-related signaling. Consistently, we detected a significant decrease in the proportion of acrosome-reacted sperm following melamine treatment under capacitating conditions. Capacitation is a tightly regulated process that relies on a coordinated sequence of events, in which cholesterol efflux initiates membrane remodeling, calcium influx sustains intracellular signaling, and downstream kinase activation drives the acquisition of fertilizing competence [30,33]. The decrease in CTC-positive cells observed here therefore points to a disruption of calcium-dependent signaling. This interpretation is reinforced by the melamine-induced increase in cholesterol efflux detected even under non-capacitating conditions. Under physiological circumstances, cholesterol efflux represents one of the earliest and most tightly controlled steps of capacitation, enhancing membrane fluidity and activating calcium-dependent signaling cascades that are indispensable for sperm maturation [34,35,36]. In contrast, the paradoxical increase in efflux observed in melamine-treated sperm, in the absence of functional capacitation, suggests that this compound triggers a premature or excessive release of cholesterol. Such abnormal remodeling likely decouples cholesterol efflux from the subsequent calcium influx and kinase activation, rendering the process ineffective. Instead of priming sperm for successful capacitation, melamine-induced efflux may saturate or desensitize signaling pathways, thereby preventing their proper propagation.

Finally, our study provides evidence for potential genotoxic effects of melamine; in fact, we observed an induced upregulation of the pro-apoptotic gene *BAX*, coupled with downregulation of the anti-apoptotic *BCL2*. These transcriptional changes were more pronounced under capacitating conditions, suggesting that sperm undergoing physiological modifications are particularly vulnerable to melamine-induced apoptotic stress. This observation aligns with previous studies demonstrating that capacitation increases susceptibility to oxidative and environmental insults [37]. Furthermore, the significant increase in sperm DNA fragmentation and denaturation, indicative of genotoxic stress, induced by melamine treatment may have implications for transgenerational effects. Indeed, sperm DNA integrity is essential for proper embryo development and successful pregnancy, with DNA damage being associated with fertilization failure, impaired embryo quality, and miscarriage [38,39].

The main limitation of this study lies in the in vitro setting and the reliance on an acute exposure model; as such, extrapolation to chronic, low-dose in vivo conditions should be approached with caution. While this reductionist design allows for the precise dissection of melamine-specific mechanisms, it may not fully capture the complexity of in vivo dynamics or synergistic interactions with other stressors. Consequently, future research employing complementary models—including longer-term exposure systems and clinically relevant subpopulations—will be essential to fully address the translational relevance of these findings

## 5. Conclusions

In conclusion, our findings demonstrate that melamine, even at low non-cytotoxic concentrations, disrupts key processes of sperm functional maturation. By uncoupling early membrane remodeling from calcium-dependent signaling, melamine impairs capacitation, reduces acrosome reaction, alters P-Tyr localization, and triggers mitochondrial dysfunction and apoptotic pathways. These effects highlight capacitation as a critical window of vulnerability and align with literature linking environmental toxicants to oxidative stress and reproductive impairment. Importantly, these findings raise broader concerns for male reproductive health in populations exposed to melamine, supporting the inclusion of melamine among emerging environmental contaminants of concern and informing public health policies aimed at minimizing subtle, subclinical reproductive toxicity.

## Data Availability

The original contributions presented in this study are included in the article/Supplementary Materials. Further inquiries can be directed to the corresponding author.

## Supplementary Materials

The following supporting information can be downloaded at: https://www.mdpi.com/article/10.3390/antiox15010122/s1, Table S1: Characteristics of primary and secondary antibodies used for immunofluorescence staining and Western Blots; Table S2: List of primers used for qRT–PC; Table S3: Demographic characteristics of participants.
