# Supplementary material for: Functional and Molecular Characterization of Melamine-Induced Disruption of Human Spermatozoa via Oxidative Stress and Apoptotic Pathways: An In Vitro Study
Source: Antioxidants (Basel). 2026 Jan 17;15(1):122. doi: 10.3390/antiox15010122 (PMC12837316; doi:10.3390/antiox15010122)
Supplement: Supplementary file 1 [file antioxidants-15-00122-s001.zip › antioxidants-4052306-Supplementary Materials.pdf]

Supplementary Materials

Supplementary Table S1. Characteristics of primary and secondary antibodies used for immunofluorescence staining and Western Blots.

Antigen	Donor Species	Dilution	Manufacturer	RRID
**Primary antibodies**				
P-Tyrosine	Mouse	1:50-1:250	Santa Cruz Biotechnology	AB_628123
COX-4	Rabbit	1:400	Thermo Fisher	AB_10987478
B Tubulin	Mouse	1:100	Thermo Fisher	AB_2609649
**Secondary antibodies**				
Anti-Mouse-IgG HRP	Goat	1:8000	Bio-Rad laboratories	AB_609692
Anti-Rabbit-IgG HRP	Goat	1:8000	Bio-Rad laboratories	AB_1102634
Alexa Fluor Anti-Mouse 568	Goat	1:500	Thermo Fisher	AB_1500898

Supplementary Table S2. List of primers used for qRT–PC

Gene	Acronymous	Chr. localization	Primer set
**Glyceraldehyde-3-Phosphate Dehydrogenase**	***GAPDH***	*chr12:6,534,512-6,538,374*	Fw 5′- ACATCGCTCAGACACCATG -3′<br>Rev 5′ – TGTAGTTGAGGTCAATGAAGGG – 3′
**Beta-2-Microglobulin**	***B2M***	*chr15:44,711,358-44,718,851*	Fw 5′ – GGACTGGTCTTTCTATCTCTTGTAC – 3′<br>Rev 5′ – ACCTCCATGATGCTGCTTAC – 3′
**RNA, 18S Ribosomal**	***18S***	*chr13*	Fw 5′ – GGGAGGTAGTGACGAAAAATAAC – 3′<br>Rev 5′ –TTGCCCTCCAATGGATCCT – 3′
**BCL2 Apoptosis Regulator**	***BCL2***	*chr18:63,123,346-63,320,128*	Fw 5′-GGCTGGGATGCCTTTGTG -3′<br>Rev 5′-GCCAAACTGAGCAGAGTCTTCA -3′
**BCL2 Associated X**	***BAX***	*chr19:48,954,815-48,961,798*	Fw 5′-TGGAGCTGCAGAGGATGATTG -3′<br>Rev 5′-TTGCCGTCAGAAAACATGTCA -3′
**Cytochrome C Oxidase Subunit 4I1**	***COX4I1***	*chr16:85,798,633-85,807,068*	Hs00971639_m1

Supplementary Table S3. Demographic characteristics of participants

**Semen parameters**	**Mean (SD)**
Volume (mL)	3,11 ± 1,00
pH	7,50 ± 0,14
Sperm Concentration (10^6/mL)	96,50 ± 21,32
Total sperm number (10^6)	284,1 ± 64,48
Motility (%)	64,67 ± 3,93
Vitality (%)	77,67 ± 2,65
Morphology (%)	8,50 ± 1,58
